# Inferring defense-related gene families in Arabidopsis and wheat

**DOI:** 10.1186/s12864-017-4381-3

**Published:** 2017-12-19

**Authors:** Rong-Cai Yang, Fred Y. Peng, Zhiqiu Hu

**Affiliations:** 1 Feed Crops Section, Alberta Agriculture and Forestry, 7000 - 113 Street, Edmonton, AB T6H 5T6 Canada; 2 Department of Agricultural, Food and Nutritional Science, University of Alberta, 410 Agriculture/Forestry Centre, Edmonton, AB T6G 2P5 Canada

**Keywords:** Comparative genomics, Arabidopsis, *Arabidopsis thaliana*, Gene families, Rust resistance genes, DNA markers, Single nucleotide polymorphism (SNP), Bread wheat, *Triticum aestivum*, Genome analysis

## Abstract

**Background:**

A large number of disease resistance genes or QTLs in crop plants are identified through conventional genetics and genomic tools, but their functional or molecular characterization remains costly, labor-intensive and inaccurate largely due to the lack of deep sequencing of large and complex genomes of many important crops such as allohexaploid wheat (*Triticum aestivum* L.). On the other hand, gene annotation and relevant genomic resources for disease resistance and other defense-related traits are more abundant in model plant Arabidopsis (*Arabidopsis thaliana*). The objectives of this study are (i) to infer homology of defense-related genes in Arabidopsis and wheat and (ii) to classify these homologous genes into different gene families.

**Results:**

We employed three bioinformatics and genomics approaches to identifying candidate genes known to affect plant defense and to classifying these protein-coding genes into different gene families in Arabidopsis. These approaches predicted up to 1790 candidate genes in 11 gene families for Arabidopsis defense to biotic stresses. The 11 gene families included ABC, NLR and START, the three families that are already known to confer rust resistance in wheat, and eight new families. The distributions of predicted SNPs for individual rust resistance genes were highly skewed towards specific gene families, including eight one-to-one uniquely matched pairs: *Lr21-NLR, Lr34-ABC, Lr37-START, Sr2-Cupin, Yr24-Transcription factor, Yr26-Transporter, Yr36-Kinase* and *Yr53-Kinase*. Two of these pairs, *Lr21*-*NLR* and *Lr34*-*ABC*, are expected because *Lr21* and *Lr34* are well known to confer race-specific and race-nonspecific resistance to leaf rust (*Puccinia triticina*) and they encode NLR and ABC proteins.

**Conclusions:**

Our inference of 11 known and new gene families enhances current understanding of functional diversity with defense-related genes in genomes of model plant Arabidopsis and cereal crop wheat. Our comparative genomic analysis of Arabidopsis and wheat genomes is complementary to the conventional map-based or marker-based approaches for identification of genes or QTLs for rust resistance genes in wheat and other cereals. Race-specific and race-nonspecific candidate genes predicted by our study may be further tested and combined in breeding for durable resistance to wheat rusts and other pathogens.

**Electronic supplementary material:**

The online version of this article (doi: 10.1186/s12864-017-4381-3) contains supplementary material, which is available to authorized users.

## Background

Many plant-associated pathogens impede plant growth and reproduction. In response, plants defend themselves from pathogen attack through two layers of defense [1]. The first layer is the PAMP (pathogen-associated molecule pattern)-triggered immunity (PTI), that is, plant cell surface pattern-recognition receptors (PRRs) detect PAMP elicitors. PTI is often a non-host resistance to the non-adapted pathogens. The second layer of defense is that plant resistance (R) proteins recognize specific pathogen effectors and elicit an effector-triggered immunity (ETI). While usually occupying extracellular niches, the pathogens extract the nutrients for their growth and proliferation from host cells, and the host cytoplasm and organelles which serve as important sites of molecular host-pathogen interaction. Thus, in contrast to PTI, ETI is effective against the adapted pathogens. The ETI-based recognition is mediated by a class of R proteins or effector-recognition receptors with the nucleotide-binding site -leucine-rich repeat (NBS-LRR or NLR) domains. The NLR proteins are often involved in race-specific resistance under the ‘gene-for-gene’ framework in many crop plants [2]. The problem with the use of such race-specific *NLR* genes in crop cultivars is that they have quickly become ineffective when new, more virulent races appear in the adapted pathogens [3]. For this reason, plant breeders and pathologists have focused on discovery, characterization and use of race-nonspecific genes for durable resistance. However, decades of genetic and breeding research have only been able to identify a limited number of genes in crop plants with durable and broad-spectrum resistance to pathogens (particularly rust pathogens in cereal crops). These race-nonspecific genes include those encoding ATP-binding cassette (ABC) transporter and kinase-START (steroidogenic acute regulatory [StAR] protein-related lipid transfer) proteins [4, 5].

Stem (or black), leaf (or brown) and stripe (or yellow) rusts are among the most damaging fungal diseases of wheat and other cereal crops around the world. Leaf rust is the most common of the three diseases in the Great Plains of North America [6] and more recently stripe rust has occurred more frequently in the Canadian Prairies and other parts of the Great Plains [7, 8]. Since the rust pathogens, *Puccinia graminis* f. sp. *tritici* (stem rust), *P. triticina* (leaf rust) and *P. striiformis* f. sp. *tritici* (stripe rust), are widely distributed, capable of forming new races virulent to previously resistant cultivars, able to travel long distances and develop rapidly under optimal environmental conditions, effective control of the rust diseases has been challenging. Breeding for new resistant cultivars has been the most cost-effective means of controlling the rust diseases. Consequently a large number of genes conferring resistance to leaf rust (*Lr*), stem rust (*Sr*) and yellow rust (*Yr*) have been identified in wheat cultivars with 71 *Lr*, 57 *Sr* and 53 *Yr* genes being recently catalogued [9]. A majority of these rust resistance genes are race-specific, conferring the resistance to one or a few races of a rust pathogen and these genes are known or assumed to encode the NLR proteins. However, a few other resistance genes, such as *Yr*36 and *Lr34* (=*Lr34*/*Yr18/Sr57*) encoding kinase-START and ABC proteins, respectively, are known to confer race-nonspecific resistance (i.e., resistance to most or all races of the same rust pathogen or resistance to multiple rust pathogens). There is little information about the proteins and their families beyond these few well-characterized rust resistance genes. For this reason, Peng and Yang [5] recently used primer sequences of non-SNP markers and flanking sequences of SNP markers for known *Lr* genes or QTLs for leaf rust resistance to predict candidate genes located at the same or adjacent genomic regions of wheat, but their prediction was only able to identify the candidate genes in the three gene families (*ABC, NLR* and *START*), likely due to the limited annotations of rust resistance genes across the large, complex wheat genome.

The genome of model plant Arabidopsis (*Arabidopsis thaliana*) was first sequenced in 2000 by the Arabidopsis Genome Initiative [10] and its latest version along with functional annotation of over 27,000 genes and 35,000 proteins is maintained in TAIR10 and Araport11 [11]. The Arabidopsis genome contains many polymorphic defense-related genes [12, 13], and several pathways of Arabidopsis in response to pathogen infection have been well characterized [14]. For example, some150 *NLR* genes were found in Arabidopsis and their characterization allowed for annotation of candidate disease resistance genes in humans and many flowering plants [15, 16]. It is estimated that approximately 70% of the genes associated with the development of cancers in humans have orthologs present in Arabidopsis [15].Therefore, Arabidopsis may serve as a model system for elucidating the spectrum of plant-pathogen interactions, and the knowledge of pathogen resistance mechanisms gained from this species is useful in crop-pathogen systems.

Recently, there is a growing interest in the use of bioinformatics approaches to inferring about genes of agronomic and adaptive importance in crop plants from the model plant Arabidopsis, and such inference has included flowering-related genes in wheat and barley [17], garden pea (*Pisum sativum*) [18], soybean (*Glycine max*) [19, 20], mungbean (*Vigna radiata*) [21] and cotton (*Gossypium hirsutum*; [22], and rice [23, 24]. However, little work has been done with prediction of disease resistance genes in crop plants from Arabidopsis. The recent release of wheat genome sequences [25] along with subsequent efforts in more in-depth sequencing will allow for the use of bioinformatics approaches to identifying new defense-related genes that encode the proteins of different families in addition to ABC, NLR and kinase-START families described above through comparative analyses of wheat and Arabidopsis genomes. Thus, the objectives of this study are (i) to infer homology of defense-related genes in Arabidopsis and wheat and (ii) to classify these homologous genes into different gene families.

## Methods

**Fig. 1:**
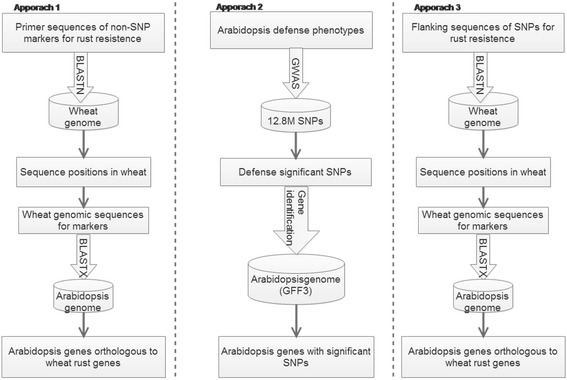
Flow charts for three approaches to prediction of candidate genes for defense-related traits in Arabidopsis. BLASTN searches a nucleotide BLAST database using a nucleotide query. BLASTX searches a protein database using a translated nucleotide query. GWAS performs genome-wide scan of associations between SNPs and defense-related traits

We employed three bioinformatics and genomics approaches (Fig. 1) to identifying candidate genes known to affect the two types of plant immunity, PTI (pathogen molecular pattern triggered immunity) and ETI (effector triggered immunity), and to classifying these protein-coding genes into different gene families in Arabidopsis. Approach 1 inferred homologous genes in Arabidopsis based on sequences of non-SNP DNA markers for rust resistance in wheat. Approach 2 directly identified SNP markers residing within or in the neighborhood (5 Kb upstream from 5′ end or downstream from 3′ end) of candidate genes from a genome-wide scan of associations between SNPs and 21 defense-related traits in Arabidopsis. Approach 3, like Approach 1, inferred homologous genes in Arabidopsis, but based on sequences of QTL SNPs for wheat rust resistance. Below we provide a detailed description of the three approaches.

### Approach 1: Prediction of homologous genes in Arabidopsis using sequences of non-SNP markers for rust resistance in wheat

We collected a set of 116 non-SNP markers linked to QTLs or genes for wheat rust resistance as reported in Liu et al. [26] and other sources. These markers were developed for mapping of 54 leaf (*Lr*), stem (*Sr*) and yellow (*Yr*) rust resistance genes (Additional file 1). We found their primer sequences in GrainGenes [27], MASWheat (http://maswheat.ucdavis.edu) and the literature [28, 29]. To identify the genomic or scaffold regions surrounded by these markers using BLASTN [30], we generated each query sequence by concatenating the forward and reverse primers of a primer pair, with five ‘N’ letters inserted between them as gaps. The query sequences were then used to search against the wheat genome sequence assembly (Release 34; Triticum_aestivum.TGACv1.dna_sm.toplevel.fa) downloaded from Ensembl Plants [31]. Because each primer query is usually less than 50 bp in length, blastn-short, a BLASTN program optimized for sequences shorter than 50 bases [30], was used with settings of a word size of seven and a relaxed E-value of 100. For each primer pair, we retained up to six best hits, taking into account genomic duplications in allohexaploid wheat in which there are three closely related genomes (A, B, and D). Based on the positions of scaffolds matching the primers in the BLAST output, we extracted the scaffold subsequences covered by these primers. To speed up subsequence extraction, we first indexed all the sequences in the large wheat genome assembly (including 735,943 scaffolds for a total of ~13 Gb of accessible wheat genomic sequences) with SAMtools [32]. Finally, we identified the Arabidopsis homologous genes using BLASTX (BLAST with translated query DNA sequences; E-value cutoff of 1e-5) to search the wheat scaffold subsequences against the newly re-annotated proteome sequences downloaded from Araport11 [11].

### Approach 2: Analysis of single SNP association with defense-related phenotypes in Arabidopsis

We [33] recently used the phenotype data for 23 flowering, 23 defense-related, 18 ionomics and 43 developmental traits and 250 K SNP markers assayed for 199 inbred lines of *A. thaliana* as described in Atwell et al. [34] for estimation of heritability using a marker-based linear mixed model analysis. Here, we used the same phenotype data for 23 defense-related traits, but took advantage of more genotype data of ~12.8 million SNP markers recently released by the 1001 Arabidopsis genomes project [35]. The variant annotated SnpEff VCF file consisting of 1135 accessions (inbred lines) genotyped for 12,883,854 SNPs was downloaded from http://1001genomes.org/data/GMI-MPI/releases/v3.1/1001genomes_snpeff_v3.1/. A R program (Additional file 2) was written to read this huge VCF file by parts (chunks), convert the VCF format for genotype coding into a numeric format (0, 1 and 2) and save each part of the data into a separate text file. Additionally, after removing those SNPs with more than two alleles and matching with the subset of *n* (<199) inbred lines for each of the defense-related phenotypes, a total of polymorphic markers were considerably reduced to a range of 2.4–3.7 million SNPs per trait.

Of the 23 defense-related traits, the two trichome-related traits were excluded from further analysis as trichomes are just morphological characteristics more related to plant defense to abiotic stresses (like waxes or thorns). The remaining 21 phenotypes represent the responses of Arabidopsis against three different types of plant pests: two bacteria (*Pseudomonas syringae* and *P. viridiflava*), a fungus (*Peronospora parasitica*) and an herbivorous insect (*Myzus persicae*) (Additional file 3). The 21 defense-related traits consisted of 12 quantitative (continuously varying) traits and nine binary (e.g. disease presence or absence) traits. For a quantitative trait, its phenotype values were first grouped according to the two possible homozygotes at each SNP locus for individual inbred lines, and the *p*-value from the *t*-test for the equality of the means of the two groups was then recorded for the SNP marker. The raw *p*-values were adjusted using the false-discovery rate (FDR) method [36] to control the false-positive rate. A significant SNP was declared if the adjusted *p*-value is less than 0.01. For a binary trait, a 2 × 2 contingency table with the two homozygotes at each SNP in the rows and two phenotypes in the columns was first constructed, and the *p*-value from the Fisher’s exact test for no association between genotypes and phenotypes (i.e., independence of rows and columns) was then calculated. However, we did not apply the FDR correction for the binary traits because Fisher’s exact test would give non-uniform *p*-values across all SNPs. The *t*-test, Fisher’s exact test, and the FDR adjustment for *p*-values were performed using R package ‘stats’ version 3.2.2 [37] (see Additional file 2 for R codes).

Recorded in the tab of “AT_sig_SNPs_by_chr” of Additional file 3 are the numbers of SNPs that were significantly (*P* < 0.01) associated with each of the 21 defense-related traits based on the above genome-wide scan for SNP-trait associations. There were 712,495 significant SNPs after summing over all 21 traits, but only 618,730 of them were found unique as some SNPs were significantly associated with multiple traits. We then found the candidate genes containing at least one of the unique SNPs, using the chromosomal positions of these SNPs and those of the genes in the Arabidopsis genome annotation file (Araport11_GFF3_genes_transposons.201606.gff). This GFF (generic feature format) annotation file was obtained in Araport11 [11]. To further reduce the possibility of false associations of SNPs and defense-related traits (in addition to the above FDR adjustment for individual SNP-trait associations), we only used candidate genes containing at least one SNP significantly associated with more than two disease phenotypes for subsequent analyses. This more stringent inclusion of SNPs resulted in only 6393 candidate genes as shown in the tab “AT_candidate genes” of Additional file 3.

### Approach 3: Inferring homologous genes in Arabidopsis from QTL SNP markers for wheat rust resistance

The data sets consisting of mapped SNPs for rust resistance at seedling or adult stage were taken from the “GWAS Results” in the Triticeae Toolbox (T3) database (https://triticeaetoolbox.org/wheat/qtl/qtl_report.php) [38]. By selecting ‘Biotic stress’ in the “Category” column and by clicking appropriate rust traits at adult or seedling stage in the “Traits” column, the T3 would display the results from the GWAS analysis for associations between markers (including Infinium 9 K, Infinium 90 K, and GBS restriction sites) and traits for individual trials (individual locations or inoculum types) within the T3 database (Additional file 4). The GWAS analysis for detection of significant SNPs was carried out using rrBLUP GWAS package [39] for individual trials or the combined analysis across all trials with the genotype-by-environment interaction effect being adjusted by including those principle components that accounted for more than 5% of the environment-relationship matrix variance as fixed effects in the mixed-model analysis. The sequences of the significant SNPs from the GWAS analysis were obtained in T3 and CerealsDB [40]. The genomic or scaffold regions surrounded by these SNP markers were inferred following Approach 1.

### Classification of the Arabidopsis proteins into different protein families

For the protein-coding genes identified above, we found their protein sequences in Araport11 and classified them into different protein families. The classification was carried out with hmmscan in the HMMER package [41] and the HMM (hidden Markov model) profiles of different protein families in the Pfam (v31.0) database [42]. To classify more than 8000 protein sequences, we installed a standalone version of HMMER (v3.1b2) from http://hmmer.org, and downloaded the HMM database from the Pfam FTP site (ftp://ftp.ebi.ac.uk/pub/databases/Pfam/releases/Pfam31.0/). This Pfam release includes a total of 16,712 protein families and 604 clans (superfamilies). To accelerate profile searches with hmmscan, the Pfam HMM database (as a flat file named Pfam-A.hmm) was first compressed and indexed with hmmpress, a software tool also included in HMMER. The E-value was set to 1e-5 in the hmmscan classification, and its output was used to classify the proteins into different families and clans based on clan memberships and their descriptions available on the Pfam FTP site.

## Results

### Prediction of protein-coding resistance genes in Arabidopsis

**Fig. 2:**
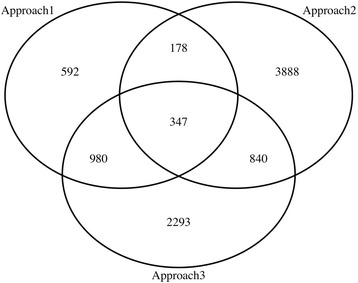
Venn diagram for numbers of defense protein-coding genes and overlaps between the three approaches. Totals of 2097 (Approach 1), 5253 (Approach 2) and 4460 (Approach 3) predicted candidate genes are overlapped with each other

The three approaches predicted different numbers of resistance genes in Arabidopsis (Fig. 2), but partial overlaps were evident between the approaches. In the first approach, a total of 2097 Arabidopsis homologous genes were identified from searching the wheat scaffold subsequences surrounded by the 116 rust resistance non-SNP markers in winter wheat (Additional file 1) obtained from Liu et al. [26] against the newly re-annotated proteome sequences downloaded from Araport11 [11]. The second approach directly predicted 5970 protein-coding (5253 with domains and 717 without domains) and 425 noncoding genes based on sequences of significant SNPs (a total of 6393 candidate genes in the tab “AT_candidate genes” of Additional file 3) from the genome-wide scan of associations between SNPs and 21 defense-related traits in Arabidopsis. A predicted gene showed significant (*P* < 0.01) associations with up to six defense-related traits. Only 525 of these candidate genes were found to have a partial to full overlap of their sequences with those of the candidate genes inferred from sequences of QTL markers for wheat rust resistance by Approach 1 (Fig. 2). In the third approach, a total of 4460 Arabidopsis homologous genes were identified from searching the wheat scaffold subsequences surrounded by 2077 rust resistance SNPs in the T3 database against the newly re-annotated proteome sequences downloaded from Araport11(Additional file 4). The number of predicted resistance genes shared by different approaches varied with 347 genes being shared by all three approaches, 525 genes shared by approaches 1 and 2, 1327 genes shared by approaches 1 and 3 and 1187 genes shared by approaches 2 and 3.

**Fig. 3:**
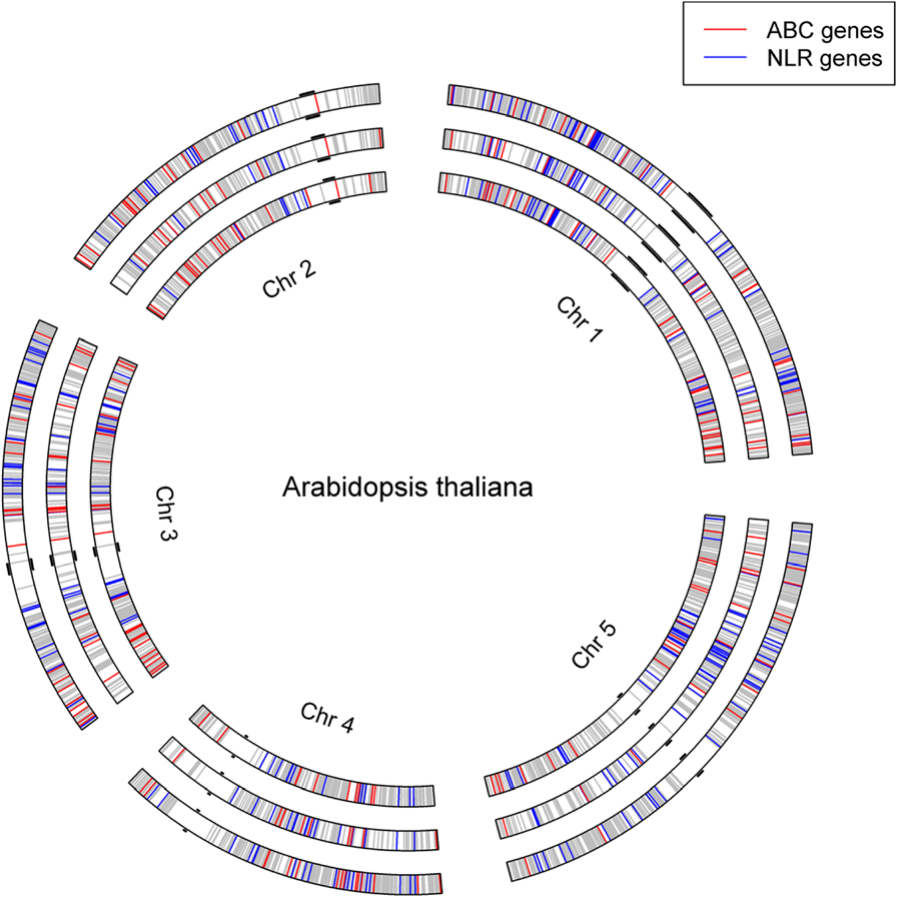
Distribution of candidate genes in 11 gene families for plant defense on five chromosomes of the Arabidopsis genome as revealed by the three approaches. The circles were arranged according to the three approaches: inner, middle and outer circles for Approaches 1, 2 and 3, respectively. The red, blue and grey lines on each of the three circles represent individual candidate genes in *ABC*, *NLR* and other nine (*START, Kinase, Transcription factor, Transporter, Cupin, Peroxidase, Protease P450* and *Tetratricopeptide repeat*) gene families, respectively. The centromeric region is marked by black bumps on each chromosome

Genomic positions of the predicted candidate genes in the 11 gene families by the three approaches are given in Fig. 3. From colored gene positions for the ABC (red) and NLR (blue) families, we noted a tendency of the genes within each family being clustered together over genomic regions on the Arabidopsis chromosomes. Lack of genes near the centromeric regions as shown in Fig. 3 is consistent with the well-known belief that most centromeres are at the gene-poor regions with inactive and repetitive constitutive heterochromatin domains [43]. The centromeric regions marked in Fig. 3 were somewhat arbitrary as they were simply marked by the two known genes as the nearest neighbors of the centromeres located the short-arm and long-arm of each Arabidopsis chromosome [44].

Of all protein-coding genes predicted by the three approaches described above, some had no domains, judging from the e-value cutoff of 1e-5 in hmmscan. There were 11 no-domain genes predicted by Approach 1, 717 no-domain genes by Approach 2 and 34 no-domain genes by Approach 3. The predicted protein-coding genes without domains were excluded from further analysis because they could not be classified into any gene family which is domain-specific. Approaches 1 and 3 were unable to find noncoding genes because we used the Arabidopsis proteome database in the final BLAST search (BLASTX). In contrast, Approach 2 allowed for predicting the presence of 425 genes outside protein-coding ORFs (open-reading frames) across the Arabidopsis genome (Additional file 3). These noncoding genes included various types of RNAs: antisense RNA, long-noncoding RNA, small nucleolar RNA, novel transcribed region, microRNA (miRNA), pre-tRNA, small nuclear RNA, antisense long-noncoding RNA and other RNA.

### Predicted gene families

There were 11 major families of genes for Arabidopsis defense against biotic stresses (diseases and insects) as uncovered by three approaches (Table 1). Three of these families, ABC, NLR and START, are already known to confer race-specific and race-nonspecific resistance to rusts in wheat and other cereal crops [4, 5]. The remaining eight families were now found to be associated with Arabidopsis defense against pests. Actually, the transcription factor superfamily was a composite group consisting of five protein families (bHLH, bZIP, Homeobox, MYB and WRKY) with known relevance to biotic and abiotic stresses. An additional category “Others” included various families each with a small number of genes or no clan (superfamily) information in the Pfam database.

**Table 1:** The number of predicted candidate genes within 11 gene families for defense-related traits in Arabidopsis

Protein family	Approach 1	Approach 2	Approach 3	Genome-wide total
ABC	132	112	91	233
NLR	225	177	171	333
START	9	13	9	36
Kinase	406	372	867	1307
TF^a^	205	123	72	683
Transporter	53	38	65	138
Cupin fold	123	68	88	251
Peroxidase	73	21	76	84
Protease	117	72	72	272
P450	109	58	210	250
TPR^b^	338	254	507	931
Others	456	4066	2242	18,969
*Total*	*2246*	*5374*	*4470*	*23,487*

^a^TF, Transcription factor superfamily including bHLH, bZIP, Homeobox, MYB, WRKY

^b^ TPR, Tetratricopeptide repeat superfamily

While Approach 2 predicted more genes than the other two approaches, the majority (4066/5374) were in the “Others” category including various families each with few genes or no clan (superfamily) information in the Pfam database. Consequently, Approaches 1 and 3 had more predicted genes in most of the 11 protein families than Approach 2. It should also be pointed out that the total numbers of predicted genes in Table 1 did not match the totals given in the Venn diagram (Fig. 1). This is because a protein-coding gene with possible multiple domains might be assigned to more than one family after Pfam classification. The genome-wide totals for individual families were obviously not the simple sums of the numbers over the three approaches, but rather they were obtained using the Arabidopsis proteome sequences and the same classification method as for the individual approaches.

### Associations between defense-related phenotypes in Arabidopsis and protein families

There were obvious associations between defense-related phenotypes and protein families in Arabidopsis (Table 2; Additional file 5). For example, Aranzana et al. [45] and Atwell et al. [34] observed hypersensitive responses of Arabidopsis seedlings (leaf collapse) when the seedlings were inoculated with the four transformed strains of bacterial pathogen, *Pseudomonas syringae*: PstDC3000::avrPphB, Pst DC3000::avrRpm1, Pst DC3000::avrB and Pst DC3000::avrRpt2, representing the four *avr* genes, respectively, while they observed no hypersensitive response when the seedlings were inoculated with a negative control, *P. syringae* DC3000 without the *avr* genes. Based on the significant SNPs for individual hypersensitive responses to the transformed strains, we predicted a total of 25 *NLR* genes, but only five *ABC* genes. Similarly, based on the significant SNPs for non-hypersensitive response to the negative control, we predicted almost the equal numbers of *NLR* (16) and *ABC* (14) genes. These observations indicate that the hypersensitive responses would be strain-specific or race-specific (i.e., *NLR*-dominant), but the non-hypersensitive response to the negative control would not.

**Table 2:** The number of predicted genes in 11 gene families for 21 defense-related traits in Arabidopsis

Phenotype^a^	ABC	Cupin	Kinase	NLR	P450	Peroxidase	Protease	START	TPR^b^	TF^c^	Transporter	Total
AvrPphB	4	9	2	1	9	1	1	2	5	6	0	40
AvrRpm1	1	8	1	1	12	0	0	4	14	5	2	48
AvrB	1	8	1	1	12	0	0	4	14	6	2	49
AvrRpt2	0	0	0	0	1	0	0	0	1	0	1	3
DC3000	14	16	3	5	32	1	0	8	28	24	7	138
LP23.1a	13	22	0	5	18	1	0	2	19	11	4	95
RMX23.1a	40	44	4	28	92	1	1	19	89	34	20	372
RMX3.1b	8	4	0	0	6	3	1	2	5	5	1	35
PNA3.3a	4	5	0	3	9	1	0	2	8	3	2	37
ME3.1b	65	79	8	39	126	14	8	36	162	54	36	627
LP23.1a CFU2	26	37	0	21	76	12	7	20	99	38	20	356
RMX23.1a CFU2	65	81	8	50	148	13	9	37	161	68	38	678
RMX3.1b CFU2	20	37	3	14	39	5	2	11	54	30	15	230
PNA3.3a CFU2	44	48	6	16	72	5	3	22	83	33	23	355
ME3.1b CFU2	11	15	1	8	21	1	1	3	16	5	1	83
Emco5	1	1	1	0	0	0	1	0	0	2	1	7
Emwa1	3	4	1	1	3	0	1	3	6	5	3	30
Emoy2	2	1	0	0	3	0	0	2	2	2	2	14
Hiks1	3	4	1	2	6	0	0	3	8	4	4	35
Noco2	1	2	0	1	5	0	0	2	5	2	0	18
Aphid number	10	6	0	5	4	0	0	1	8	4	1	39

^a^ See Additional file 2 for detailed description of 21 defense-related phenotypes

^b^TF, Transcription factor superfamily including bHLH, bZIP, Homeobox, MYB, WRKY

^c^ TPR, Tetratricopeptide repeat superfamily

On the other hand, the numbers of predicted genes with resistance to another bacterial pathogen, *P. viridiflava*, were much more abundant than to *P. syringae*, particularly those genes encoding proteins in ABC, NLR, Cupin, Kinase, P450, TPR, TF and transporter families (Table 2; Additional file 5). The predicted genes were more evenly distributed over these protein families as well. Unlike *P. syringae* and most other plant pathogens, *P. viridiflava* is capable of infecting a large number of host species including the model plant *A. thaliana* [46, 47]. In particular, *P. viridiflava* is not subdivided into host-specific strains or races as is *P. syringae*. Such a wide range of hosts by *P. viridiflava* may arise from its unique characteristics including (i) being an opportunistic pathogen that causes severe disease only with favorable environmental conditions and (ii) being an epiphyte that is abundant in its hosts but without associated disease symptoms [47].

The numbers of predicted genes with resistance to downy mildew caused by five strains of the biotrophic fungal pathogen, *Peronospora parasitica* [34, 48] were too few to uncover any meaningful patterns across different protein families (Table 2; Additional file 5). Nevertheless, the numbers of significant SNPs were fewer within predicted genes with resistance to this fungal pathogen than to the two bacterial pathogens described above even though the percentages of the total SNPs were well within the ranges for all the pathogens. Similarly, the numbers of predicted genes with resistance to aphid (*Myzus persicae*) were also limited across all protein families.

### In Silico mapping of resistance on wheat and Arabidopsis genomes

The number of SNPs being significantly associated with the 21 defense traits in Arabidopsis varied among different protein families (Table 3; the tab of “defense_related SNPs” in Additional file 6). The SNPs were annotated according to their physical positions in the following genomic regions: upstream, 5’ UTR, coding (missense, synonymous), intron, stop codon, 3’ UTR and downstream. Such detailed annotation revealed further insights into the distributions of the significant SNPs over coding and noncoding genomic regions. For example, focusing on the two gene families, *NLR* and *ABC*, known to be associated with race-specific and race-nonspecific resistance to wheat rusts, respectively, we observed that the SNPs in the *NLR* genes were more abundant than those in the *ABC* genes over all genomic regions with an obvious exception of intronic regions. It is also of interest to note that the hypersensitive response to the transformed strain AvrRpt2 of *P. syringae* was the only trait with more SNPs in the coding (missense and synonymous) regions of the *ABC* genes than in the coding regions of the *NLR* genes whereas the reverse pattern was true for all the other traits.

**Table 3:** The total number of SNPs in coding and non-coding regions of predicted genes in 11 gene families for 21 defense-related traits in Arabidopsis

Phenotype^a^	ABC	Cupin	Kinase	NLR	P450	Peroxidase	Protease	START	TPR^b^	TF^c^	Transporter	Total
AvrPphB	1948	868	4004	3253	1001	70	614	285	3000	1423	1169	17,635
AvrRpm1	3635	1223	6539	5513	1493	331	313	207	4638	1687	1549	27,128
AvrB	1839	357	4331	3170	1048	70	551	285	2722	1351	886	16,610
AvrRpt2	2153	304	3915	2021	911	191	211	267	2190	559	698	13,420
DC3000	5325	2430	9775	7387	1532	601	643	400	7559	3113	2751	41,516
LP23.1a	4860	1908	8221	5641	1598	260	444	196	8295	3200	2155	36,778
RMX23.1a	5538	2918	11,130	7288	2586	882	711	309	9425	3857	1908	46,552
RMX3.1b	7567	3850	17,546	11,375	3990	979	1159	696	13,631	5451	4076	70,320
PNA3.3a	4989	1546	6907	5570	920	308	278	373	5271	2309	1276	29,747
ME3.1b	4653	1634	8436	5870	1926	321	673	76	4884	2038	1899	32,410
LP23.1a CFU2	6753	3214	13,341	8767	3201	880	786	487	10,726	4414	3203	55,772
RMX23.1a CFU2	6844	2944	13,110	8242	1925	440	864	570	9789	3679	2514	50,921
RMX3.1b CFU2	7605	4194	18,400	11,286	3755	1019	1159	534	14,364	5946	4139	72,401
PNA3.3a CFU2	5133	2083	8710	6282	2163	533	405	195	5865	2762	1673	35,804
ME3.1b CFU2	5460	2381	10,892	7700	2228	489	706	423	8447	3650	2623	44,999
Emco5	1872	859	3848	2084	571	133	202	345	2721	945	494	14,074
Emwa1	1117	586	2910	2355	452	0	313	138	1515	715	978	11,079
Emoy2	2267	586	3615	4376	1085	114	464	234	2435	1360	1049	17,585
Hiks1	2394	717	5451	4517	744	339	425	344	3614	1053	1193	20,791
Noco2	1610	386	2869	2948	798	82	202	57	1634	915	444	11,945
Aphid number	3471	1809	5839	4195	551	120	667	301	5456	1933	1688	26,030

^a^See Additional file 2 for detailed description of 21 defense-related phenotypes

^b^TF, Transcription factor superfamily including bHLH, bZIP, Homeobox, MYB, WRKY

^c^ TPR, Tetratricopeptide repeat superfamily

The homologous wheat sites of Arabidopsis SNPs (Table 4; the tab of “rustmarker_gene SNPs” in Additional file 6) tended to be present in specific gene families, depending on whether rust resistance genes are race-specific or race-nonspecific. For example, all predicted SNPs residing around the neighborhood of *Lr21*, a known race-specific *R* gene, were present only in the NLR family across all coding and noncoding regions. On the other hand, all the predicted SNPs residing around the neighborhood of *Lr34*/*Yr18/Sr57*, a known race-nonspecific rust resistance gene, were present only in ABC family across all genomic regions. Similarly, all the predicted SNPs residing around the neighborhood of *Sr2/Lr27*, another known race-nonspecific rust resistance gene, were present only in the cupin family across all genomic regions. Since the protein encoded by *Yr*36 contains a kinase domain and a START domain [49], all the predicted SNPs residing around the neighborhood of *Yr*36 appeared in the kinase family as we assigned the *Yr*36-associated SNPs to this family rather than to the START family.

**Table 4:** The number of homologous SNPs in coding and non-coding regions of rust resistance genes for 11 gene families

Rust resistance^a^	ABC	Cupin	Kinase	NLR	P450	Peroxidase	Protease	START	TPR^b^	TF^c^	Transporter	Total
*Lr17a*	0	0	0	4237	0	0	0	0	0	33,270	0	37,507
*Lr21*	0	0	0	2275	0	0	0	0	0	0	0	2275
*Lr27*	0	0	490,734	193,899	0	0	0	0	0	0	0	684,633
*Lr34*	32,659	0	0	0	0	0	0	0	0	0	0	32,659
*Lr37*	0	0	0	0	0	0	0	5530	0	0	0	5530
*Lr46*	0	2482	0	0	0	0	0	0	0	2417	0	4899
*Lr60*	606	0	0	90,923	0	0	0	0	0	0	0	91,529
*Lr68*	18,963	0	0	27,110	0	0	0	8943	0	15,209	0	70,225
*QSr.abr-*	70,003	0	0	29,571	108,568	0	0	0	0	0	0	208,142
*Sr13*	2169	0	0	3760	0	0	0	0	0	0	0	5929
*Sr2*	0	74,738	0	0	0	0	0	0	0	0	0	74,738
*Sr22*	0	0	1077	0	114,579	0	0	0	459,695	0	0	575,351
*Sr26*	4036	155,936	0	0	0	0	118,953	0	0	0	0	278,925
*Sr35*	0	0	3836	0	0	0	0	0	0	0	0	3836
*Sr36*	0	0	0	0	0	109,350	0	0	0	192,982	0	302,332
*Sr39*	0	0	63,540	0	0	0	0	0	1342	0	0	64,882
*Sr43*	93,131	0	0	12,242	0	0	0	0	0	0	0	105,373
*Sr45*	1647	0	0	0	0	0	0	0	0	5225	0	6872
*Sr56*	0	0	1923	0	0	0	0	0	0	4278	0	6201
*Yr24*	0	0	0	0	0	0	0	0	0	21,030	0	21,030
*Yr26*	0	0	0	0	0	0	0	0	0	0	110,296	110,296
*Yr36*	0	0	4957	0	0	0	0	0	0	0	0	4957
*Yr53*	0	0	4250	0	0	0	0	0	0	0	0	4250
GSI^d^	0.698	0.450	0.247	0.640	0.500	0.000	0.000	0.472	0.006	0.481	0.000	

^a^Some genes are known to confer resistance to multiple rusts and other pathogens and they include Lr34 (=Lr34/Yr18/Bdv1/Pm38/Ltn1), Lr37(=Lr37/Sr38/Yr17), Lr46(=Lr46/Yr29/Pm39/Ltn), Sr2(=Sr2/Lr27/PBC/Pm) and Sr39(=Sr39/Lr35)

^b^TF, Transcription factor superfamily including bHLH, bZIP, Homeobox, MYB, WRKY

^c^ TPR, Tetratricopeptide repeat superfamily

^d^GSI = Gini-Simpson diversity index

## Discussion

This study employed three bioinformatics and genomics approaches to predict up to 1790 defense-related candidate genes within 11 gene families (Table 1) in Arabidopsis and their homologs for race-specific and race-nonspecific resistance to leaf, stem and stripe rusts in wheat (Table 4). In addition to the three gene families (*ABC*, *NLR* and *START*) that are already known to confer race-specific and race-nonspecific resistance to wheat rusts and other pathogens [4, 5], eight new gene families for plant defense are now being inferred by the three approaches. It is somewhat surprising that Approach 1 (inference based on sequences of markers for wheat rust resistance) predicted more candidate genes in individual families over the Arabidopsis genome than did Approaches 2 and 3 except for the kinase family where Approach 3 predicted the most genes. Approach 2 did predict the most candidate genes overall as expected because it was based on a genome-wide scan of associations between 21 defense-related traits and 12.8 million SNPs across the Arabidopsis genome. However, the majority (>75%) of the predicted candidate genes by Approach 2 could not be assigned to any of the 11 gene families, comparing to the proportions of unassigned candidate genes being 20.3 and 50.2% by Approaches 1 and 3, respectively. The prediction was based on the latest Arabidopsis genome annotation file in Araport11 [11].

It should be of little surprise to observe partial overlaps of candidate genes for plant defense in Arabidopsis as predicted by the three approaches (Fig. 2). First of all, while all inferred candidate genes are related to plant defense, they are derived from different sources, probably representing different parts of genomic regions. The candidate genes by approaches 1 and 3 were inferred indirectly through homology between DNA sequences of Arabidopsis and wheat as theses inferences were based on the primer sequences of non-SNP (approach 1) or SNP (approach 3) markers related to rust resistance in wheat. In contrast, approach 2 inferred the candidate genes based directly on their physical positions (as shown in the Arabidopsis genome annotation file: Araport11_GFF3_genes_transposons.201606.gff [11]) relative to the chromosomal positions of the SNPs with significant associations with the 21 defense-related phenotypes. Second, the 21 phenotypes used in approach 2 covered a wide range of pests including infections of bacteria, fungi and insects whereas the phenotypes used in approaches 1 and 3 were limited to responses to three rust fungi. As genes known to be exclusively crucial for defense against bacterial intruders may not be the same for the resistance to fungal pathogens [50], the observed partial overlaps of identified candidate genes between the three approaches would likely be expected. Third, despite the evolutionary conservation of core (shared) component genes for fungal resistance in monocot (e.g., cereals including wheat) and dicot (Arabidopsis) plants since their split ~200 Mya ago, clade/species-specific innovation genes are also required to form a fully functional module in plant innate immunity or defense [50], again likely leading to the observed partial overlaps.

Another important finding from this study is that the distributions of predicted SNPs for individual rust resistance genes were highly skewed towards specific gene families (Table 4). At the extreme, the predicted SNPs for a rust resistance gene appeared only in one gene family with eight such unique association pairs being *Lr21-NLR, Lr34-ABC, Lr37-START, Sr2-Cupin, Yr24-Transcription factor, Yr26-Transporter, Yr36-Kinase* and *Yr53-Kinase*. Two of these pairs, *Lr21*-*NLR* and *Lr34*-*ABC*, are expected because *Lr21* and *Lr34* are well known to confer race-specific and race-nonspecific resistance to leaf rust and they encode NLR and ABC proteins, respectively [4, 5]. *Yr36* encodes a protein with a kinase domain fused to a putative START lipid-binding domain [49] and thus the *Yr36*-*Kinase* pair is expected though *Yr36* is sometimes cited as a gene encoding the START protein in the past [5]. The *Sr2*-*Cupin* is probably expected as well because the *Sr*2 region consists of 10 cupin-domain-containing GLP (Germin-Like Protein) genes [51]. The information is yet available on molecular and functional characterizations of the remaining four unique pairs, *Lr37*-*START*, *Yr24*-*Transcription factor*, *Yr26*-*Transporter* and *Yr53*-*Kinase*, and this is certainly an area for future research.

Functional (rust resistance) diversity of a gene family may be indicated by the predicted numbers of SNPs residing around the rust resistance genes. At one end of the diversity spectrum, three gene families were specific to single rust resistance genes (Table 4): the Peroxidase is specific to *Sr*36, the protease specific to *Sr26* and the transporter specific to *Yr26*. At the other end of the diversity spectrum, the ABC and NLR were functionally diverse as the abundant SNPs in these families were distributed over many rust resistance genes. This pattern of functional diversity was confirmed by the estimates of Gini-Simpson diversity index [52] for individual gene families: the Peroxidase, protease and transporter families were the least diverse with the estimates of zero while the ABC and NLR families the most diverse with the estimate of 0.698 and 0.640, respectively. The moderate-sized estimate of functional diversity (0.450) for the cupin family is hardly surprising as the GLPs in the cupin family are a group of small (~220 amino acid residues), functionally and taxonomically diverse proteins with two of them known to hydrogen peroxide, a plant defense signal [53]. Thus, the richness and evenness of the predicted SNPs within individual gene families may serve as a useful indicator of functional diversity of genes for rust resistance and other agronomic traits in future studies.

Our study is unique in several ways. First, our identification of the candidate genes and SNPs in coding and noncoding regions over the 11 gene families contributes significantly to current understanding of functional diversity with rust resistance genes in wheat. It was recently reported [54] that only six race-specific wheat rust resistance genes (*Lr1, Lr10, Lr21, Sr33, Sr35* and *Yr10*) were cloned, all encoding the same class of proteins with NLR domains. Even fewer (only three) race-nonspecific rust resistance genes were cloned so far [4], with *Lr34* encoding a protein in the ABC family, *Lr67* encoding a protein in the STP (sugar transporter protein) family and *Yr36* encoding a protein in the kinase family. In this study, we were able to identify up to 1790 candidate genes encoding proteins belonging to the 11 families that are in physical proximity to the rust resistance genes distributed over different wheat chromosomes. While the race-specific resistance is often due to a single *NLR* gene, there are cases (e.g., *Lr10*) where such resistance is due to two or more adjacent genes [55]. Second, our comparative genomic analysis between Arabidopsis and wheat largely avoids several problems, such as tediousness, low marker density and limited recombination rate, that often arise from the conventional map-based approaches [56, 57], thereby accelerating the discovery of rust resistance genes in wheat and other cereals. Despite the ongoing international efforts, the full genome sequence of allohexaploid wheat remains difficult to obtain because of (i) its colossal size (17.1 Gb vs. 0.135 Gb of the *Arabidopsis thaliana* genome, a 126-fold difference); (ii) high sequence identity of homologous genes of three highly related subgenomes (A, B and D); (iii) genomic complications [~24% of the genes undergoing intrachromosomal duplications and ~81% of the genome consisting of repetitive DNA, primarily LTR (long terminal repeat) retrotransposons] [58]. On the other hand, our comparative genomics approach allows for leveraging the rich genomic resources from the deeply-sequenced and well-annotated *Arabidopsis thaliana* genome [35] for identification of wheat rust resistance gene homologs. Third, our genome-wide approach broadens the scope of previous studies focusing only on functional and molecular characterizations of protein-coding genes in the *ABC* and *NLR* families for rust resistance [4, 56]. Our analysis largely confirms such characterizations, and more importantly the new candidate genes and families serve as an important basis for future research towards their complete characterization and their use for wheat breeding for rust resistance.

Our study focuses on inference of gene families for plant defense in a model plant (Arabidopsis) and an agriculturally important crop (wheat). Similar inferences can be found in other model or crop plants though they often focus on specific disease loci rather than a genome-wide approach used in our study. For example, Li et al. [59] recently conducted a GWAS analysis (similar to our approach 2) to identify a natural allele of a C_2_H_2_-type transcription factor with race-nonspecific resistance to blast pathogen (*Magnaporthe oryzae*) in rice. The resistance allele (SNP33-G) differs from its susceptibility counterpart (SNP33-A) in just a single nucleotide in the promoter of the *broad-spectrum resistance Digu 1* (*bsr-d1*) gene (*LOC_Os03 g32230*), 618 base pair before the coding region. This point mutation causes reduced gene expression through the binding of the repressive MYB transcription factor, thereby inhibiting H_2_O_2_ degradation and enhancing rice blast resistance. An added novelty of this newly discovered allele is that its broad-spectrum race-nonspecific resistance does not link to yield penalty in resistant cultivars as found, for example, in *Lr34*-wheat yield tradeoff [60] and *Pigm*-rice yield tradeoff [61]. Many other studies have used an analysis (similar to our approach 2) to reveal resistance genes largely from the *NLR* or *ABC* family in different cereals (see Table 1 of Krattinger and Keller [62] for specific examples), but it remains to be seen the interspecies homology analysis similar to our approach 1 or 3.

The knowledge on a wide array of resistance candidate genes in different gene families as acquired in our and other studies will have important implications for new breeding strategies of developing durable resistance to multiple races of the same pathogens or multiple pathogens in wheat and other cereals [56, 63]. The key to success of these new breeding strategies is the ability to stack race-specific resistance genes (mostly known in the *NLR* gene family) along with race-nonspecific genes in the other gene families (e.g., those in the *ABC* family) to produce the durable resistance to wheat rusts and other cereal pathogens. The gene stacking is preferred over the conventional breeding methods of singularly deploying single race-specific resistance genes as it will avoid lack of durability of race-specific resistance due to rapid mutation or loss of recognized pathogen effectors. The candidate resistance genes identified in our and other studies need to be further tested and validated before they can be effectively used for the gene stacking. However, the current process of gene testing and validating remains slow (one gene at a time) and costly as it often uses the gene knockout approach. For most durable resistances with complex, polygenic inheritance (e.g., a cluster of 13 genes in the *NLR* family uncovered at the *Pigm* locus for rice blast resistance [61]), it will be practically feasible in near future to have a genome-wide manipulation of the relevant genes through some latest genome editing technologies [64], thereby capturing the joint contributions of all stacked genes towards the improved durable resistance.

## Conclusions

Our study was able to identify the candidate genes in three gene families known to confer rust resistance in wheat and eight new families, thereby enhancing current understanding of functional diversity with defense-related genes in genomes of model plant Arabidopsis and cereal crop plant wheat. Our sequence-based annotation and comparative genomic analysis of Arabidopsis and wheat genomes allows for genome-wide identification of candidate genes for plant defense or other traits. In contrast, the commonly used map-based or marker-based approaches only have the limited ability to discover new defense-related genes (e.g., rust resistance genes in wheat and other cereals) due to usual challenges such as tedious fine mapping, low marker density and limited recombination rate. This is particularly true for large, complex genomes of wheat and other cereal crops with incomplete and imperfect sequencing and annotation information despite the ongoing international efforts on genome sequencing in these crops. The acquisition for a wide array of resistance candidate genes in different gene families in our and other studies is an important first step towards implementing a new gene-stacking strategy that combines race-specific resistance genes (mostly known in the NLR family) with race-nonspecific genes in the other gene families to breed for the durable resistance to wheat rusts and other cereal pathogens.

## Additional files

Additional file 1: The designated rust genes and their chromosomal locations as well as primer sequences of associated molecular markers. (XLSX 94 kb)

Additional file 2: R code for large-scale genome scan of associations between SNPs and defense-related phenotypes in Arabidopsis. (TXT 8 kb)

Additional file 3: Predicted candidate genes from a large-scale genome-wide scan of associations between SNPs and 21 defense-related traits in Arabidopsis. (XLSX 733 kb)

Additional file 4: The rust resistance QTLs from the T3 database of the wheat seedlings and adult plants and the flanking sequences of their SNP markers from CerealsDB and T3 database. (XLSX 888 kb)

Additional file 5: The distributions of 11 protein-coding gene families over 21 defense-related traits in Arabidopsis. (XLSX 18 kb)

Additional file 6: The number of predicted SNPs for rust resistance in wheat and significant SNPs for 21 defense-related traits in Arabidopsis. (XLSX 50 kb)

## Abbreviations

ABC: ATP-binding cassette
ETI: Effector-triggered immunity
NLR: Nucleotide-binding site /leucine-rich repeat (NBS-LRR)
PTI: PAMP (pathogen-associated molecule pattern)-triggered immunity
QTL: Quantitative trait locus
SNP: Single nucleotide polymorphism
START: Steroidogenic acute regulatory (StAR) protein-related lipid transfer domain
